# Chromosome Painting in Neotropical Long- and Short-Tailed Parrots (Aves, Psittaciformes): Phylogeny and Proposal for a Putative Ancestral Karyotype for Tribe Arini

**DOI:** 10.3390/genes9100491

**Published:** 2018-10-10

**Authors:** Ivanete de Oliveira Furo, Rafael Kretschmer, Patrícia C. M. O’Brien, Jorge C. Pereira, Analía del Valle Garnero, Ricardo J. Gunski, Malcolm A. Ferguson-Smith, Edivaldo Herculano Corrêa de Oliveira

**Affiliations:** 1 Programa de Pós-Graduação em Genética e Biologia Molecular, Universidade Federal do Pará, Belém, Pará 66075-110, Brazil; ivanetefuro100@gmail.com; 2 Laboratório de Cultura de Tecidos e Citogenética, SAMAM, Instituto Evandro Chagas, Ananindeua, Pará 67030-000, Brazil; 3 Cambridge Resource Centre for Comparative Genomics, Cambridge CB3 0ES, UK; allsorter@gmail.com (P.C.M.O.); jorgecpereira599@gmail.com (J.C.P.); maf12@cam.ac.uk (M.A.F.-S.); 4 Programa de Pós-Graduação em Genética e Biologia Molecular, Universidade Federal do Rio Grande do Sul, Porto Alegre, Rio Grande do Sul 91509-900, Brazil; rafa.kretschmer@gmail.com; 5 Programa de Pós-Graduação em Ciências Biológicas, Laboratório de Diversidade Genética Animal, Universidade Federal do Pampa, São Gabriel, Rio Grande do Sul 97300-162, Brazil; analiagarnero@yahoo.com.br (A.d.V.G.); rgunski@yahoo.com.br (R.J.G.); 6 Faculdade de Ciências Naturais, Instituto de Ciências Exatas e Naturais, Universidade Federal do Pará, Belém, Pará 66075-110, Brazil

**Keywords:** parrots, Psittaciformes, Psittacidae, chromosome painting, phylogeny, putative ancestral karyotype

## Abstract

Most Neotropical Psittacidae have a diploid number of 2n = 70, and a dichotomy in chromosome patterns. Long-tailed species have biarmed macrochromosomes, while short-tailed species have telo/acrocentric macrochromosomes. However, the use of chromosome painting has demonstrated that karyotype evolution in Psittacidae includes a high number of inter/intrachromosomal rearrangements. To determine the phylogeny of long- and short-tailed species, and to propose a putative ancestral karyotype for this group, we constructed homology maps of *Pyrrhura frontalis* (PFR) and *Amazona aestiva* (AAE), belonging to the long- and short-tailed groups, respectively. Chromosomes were analyzed by conventional staining and fluorescent in situ hybridization using whole chromosome paints of *Gallus* *gallus* and *Leucopternis albicollis*. Conventional staining showed a karyotype with 2n = 70 in both species, with biarmed macrochromosomes in PFR and telo/acrocentric chromosomes in AAE. Comparison of the results with the putative avian ancestral karyotype (PAK) showed fusions in PFR of PAK1p/PAK4q (PFR1) and PAK6/PAK7 (PFR6) with a paracentric inversion in PFR6. However, in AAE, there was only the fusion between PAK6/7 (AAE7) with a paracentric inversion. Our results indicate that PFR retained a more basal karyotype than long-tailed species previously studied, and AAE a more basal karyotype for Neotropical Psittacidae analyzed so far.

## 1. Introduction

The order Psittaciformes comprises cockatoos, parrots, macaws, and parakeets, with approximately 350 species distributed between 84 genera. Currently, it is separated into two families—Cacatuidae, confined to Australia and proximity, and Psittacidae, which are found in tropical regions of the world and encompass most species of the order [1,2,3].

According to Wright et al. [4] their studies support a Cretaceous origin of Psittaciformes in Gondwana after the separation of Africa and the India/Madagascar block with subsequent diversification through both vicariance and dispersal. Nevertheless, Tavares et al. [5] have even suggested that the Neotropical parrots (New World parrots) shared a common ancestor with Australian parrots (59 million years ago (Mya)), much earlier, before Australia separated from Antarctica and South America, implying that ancestral parrots were widespread in Gondwanaland.

For instance, in the Neotropics, the Psittaciformes are represented by the Tribe Arini (Psittacidae, subfamily Psittacinae), with 140 species and 30 genera. Although Arini species are very diverse morphologically [1,2], they have been classified by the length of their tails—long-tailed species and short-tailed species. This criterion was initially supported by mitochondrial DNA (mtDNA) sequencing studies (12S ribosomal DNA (rDNA), 16S rDNA, and cytochrome b) in nine species of Arini [6].

The systematics of Neotropical Psittacidae are still controversial, although different types of data have been used in attempts to resolve them [5]. New phylogenetic models have been proposed, and some taxa have been relocated to other genera. For example, *Diopsittaca nobilis*, which was formerly included in the genus *Ara*, is now included in a new genus according to the evidence from morphology and mtDNA [5,7].

The most complete phylogenetic analysis of Arini has included 29 species of 25 different genera [5], grouped into three clades: parrots of the genera *Bolborhynchus* and *Nannopsitta* (clade A), amazons and allies of the genera *Amazona*, *Pionus*, *Graydidascalus*, *Pionopsitta*, *Triclaria*, *Myiopsitta*, and *Brotogeri* (clade B), and macaws, conures, and relatives in the genera *Ara*, *Primolius*, *Orthopsittaca*, *Cyanopsitta*, *Nandayus*, *Aratinga*, *Guarouba*, *Diopsittaca*, *Anodorhynchus*, *Cyanoliseus*, *Rhynchopsitta*, *Enicognathus*, *Pyrrhura*, *Pionites*, *Deroptyus*, and *Forpus* (clade C). Additionally, it has been proposed that three of the genera were not monophyletic: *Aratinga*, *Pionopsitta*, and *Amazona* [5,7]. The three major clades of Neotropical parrots originated about 50 Mya, coinciding with periods of higher sea level when both Antarctica and South America were fragmented with transcontinental seaways, and likely isolated the ancestors of modern Neotropical parrots in different regions in these continents. The diversification of the Amazons genera and allies which occurred between 46 and 16 Mya suggests, however, that parrotlets and macaws, conures, and allies may have been isolated in Antarctica and/or the southern cone of South America, and only dispersed out of these southern regions when the climate cooled and Antarctica became ice-encrusted about 35 Mya [5].

Cytogenetic studies have shown that Neotropical Psittacidae have a constant diploid number, 2n = 70, with the exception of a few species, such as *Graydidascalus brachyurus*, *Forpus xanthopterygius* and *Brotogeris versicoluru* with 2n = 64, 82, and 84, respectively [8]. Differences in chromosomal morphology led Francisco and Galetti [9] to propose that reciprocal translocations and pericentric inversions were the main mechanisms of karyotype evolution in Arini. Recent studies based on chromosome painting in three species of two different genera—*Ara macao*, *Anodorhynchus hyacinthinus*, and *Ara chloropterus*—showed that fusions and fissions also have an important role in the karyotypical diversification of Neotropical Psittacidae [10,11]. These results confirm that, despite apparent chromosomal similarity, macaws have very diverse karyotypes.

The chromosome painting with *Gallus gallus* (GGA) probes in some Neotropical Psittacidae species indicated that fissions and fusions played an important role in the karyotype evolution of Tribe Arini. Some of these associations have been found in all Psittacidae species analyzed so far—GGA1/4, GGA6/7, and GGA8/9 [11], and seem to play an important role in the karyotype evolution of Psittacidae species. However, with GGA probes, it is not possible to detect intrachromosomal rearrangements, which can be useful for phylogenetic inferences [12]. The use of probes derived from species with highly derived karyotypes has been shown to be an important tool in the detection of intrachromosomal rearrangements and the correct assignment of chromosomal segments involved in rearrangements. For instance, whole chromosome painting probes from the white hawk (*Leucopternis albicollis*) with 2n = 66 and multiple fissions involving ancestral avian syntenies, have highlighted inversions, undetected by GGA probes, as the most common rearrangements in Passeriformes and Columbiformes [13,14,15,16].

In view of the success of chromosome painting in answering some phylogenetic questions in birds [12,14,15,17], including Psittaciformes [10,11], we have analyzed the chromosome complement of two species of the Tribe Arini, *Amazona aestiva* (AAE) and *Pyrrhura frontalis* (PFR), belonging to the short- and long-tailed groups, respectively, by chromosome painting using whole-chromosome paints of *G*. *gallus* and *L*. *albicollis*. The results are considered in relation to the classification based on tail length. They show that different rearrangements are present in species of the Tribe Arini, which help in understanding phylogenetic relationships, and suggest a putative ancestral karyotype for Neotropical Psittacidae.

## 2. Material and Methods

### 2.1. Samples, Cell Culture, and Chromosome Preparation

Experiments were approved by the ethics committee (CEUA—Universidade Federal do Pará) under no. 170/2013. The sample included two females of *Amazona aestiva* (AAE) and one *Pyrrhura frontalis* (PFR) male and female (Table S1). Tissue cell cultures from skin biopsies or feather pulp, performed according to Sasaki et al. [18], with modifications, were used to make chromosome preparations. For harvesting, we used a protocol including colcemid treatment (0.05% for 1 h), followed by incubation with hypotonic solution (KCl 0.075 M) and fixation. For the determination of diploid number and chromosome morphology, slides were stained with Giemsa (5% in phosphate buffer pH 6.8) and analyzed with 100× objective. A minimum of 20 metaphases per individual were photographed and karyotyped using Genasis software (Applied Spectral Imaging, Carlsbad, CA, USA).

### 2.2. Fluorescent In Situ Hybridization

FISH experiments were performed according to de Oliveira et al. [13], using whole-chromosome probes from *Gallus gallus*, GGA (pairs 1–10), and *Leucopternis albicollis*, LAL (pairs homologous to GGA1 (LAL 3, 6, 7, 15, and 18), 2 (LAL 2, 4, and 20), 3 (LAL 9, 13, 17, and 26), 4 (LAL 1 and 16), 5 (LAL 5), 6 (LAL 3), obtained by flow cytometry at the Cambridge Resource Centre for Comparative Genomics (Cambridge, UK). Probes were labeled with biotin or fluorescein by DOP-PCR. Chromosomes were counterstained with DAPI. Slides were analyzed, and metaphases were photographed using a Zeiss-Axiophot microscope with a 60× objective and modulated by software Axiovision version 4.1. Chromosome mapping and comparisons were based on the putative ancestral avian karyotype (PAK), in which pairs PAK 1-11 correspond to GGA1-GGA3, GGA4q, GGA5-GGA9, GGA4p, and GGA10 [19].

### 2.3. Phylogenetic Analysis

In order to construct the phylogenetic tree and clarify the phylogenetic position of some species in this group, as well as to propose a putative ancestral karyotype for Neotropical Psittacidae (PAK-NP) (Tables S2 and S3), we considered the cytogenetic information for species of Psittaciformes, taking into account the presence or absence of chromosomal characters as described by Dobigny et al. [20]. We gave special importance to chromosomal rearrangements which correspond to common features in Psittaciformes, such as associations PAK 1/4 (GGA1/4), PAK 6/7 (GGA6/7), and PAK8/9 (GGA8/9). In addition, we considered data from chromosome morphology descriptions of 31 species belong to 14 genera of the Arini Tribe.

## 3. Results

### 3.1. Karyotype Analysis

**Figure 1:**
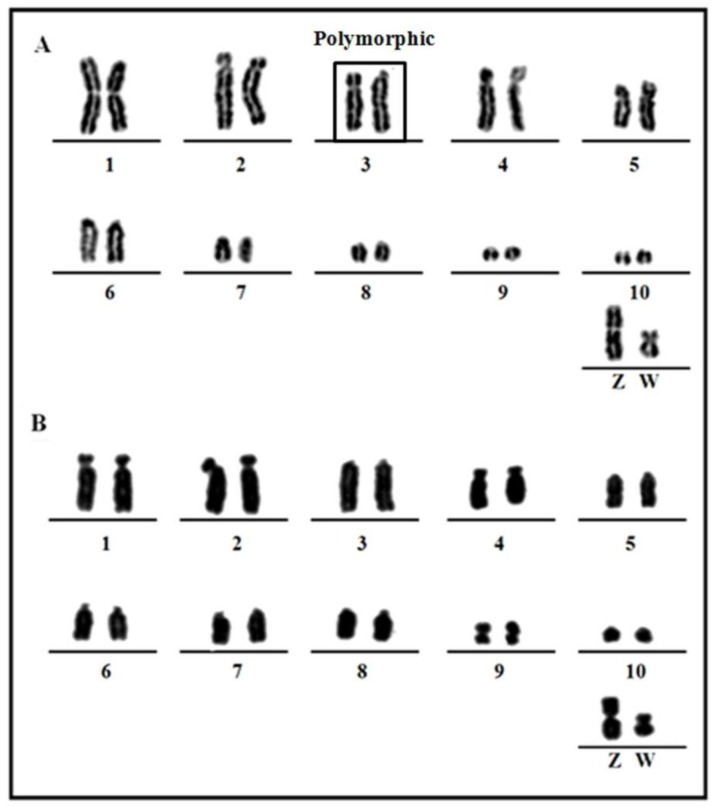
Partial karyotypes conventionally stained of the two species: (**A**) *Pyrrhura frontalis*, 2n = 70, (**B**) *Amazona aestiva* 2n = 70. Heteromorphic chromosome pair is boxed.

We found a 2n = 70 karyotype in *P*. *frontalis*. Pairs 1 and 2 were very similar in size; however, pair 1 is metacentric and pair 2 is acrocentric. Pair 3 was found to be heteromorphic in both individuals analyzed (Figure 1A), and pairs 4 and 5 were acrocentric. The remaining autosomal pairs were telocentric. The Z was metacentric and the W submetacentric, corresponding in size to the 5th and 7th pairs, respectively.

*Amazona aestiva* had a 2n = 70 karyotype, as described previously [21,22]. Pairs 5, 6, 7, and 8 were telocentric; pairs 1, 2, and 4 submetacentric; pair 9 metacentric; and pairs 3 and 10 acrocentric. Both Z and W chromosomes were metacentric, but the Z chromosome corresponded in size to the 4th pair, and W to the 9th pair (Figure 1B).

### 3.2. Comparative Chromosome Painting

**Figure 2:**
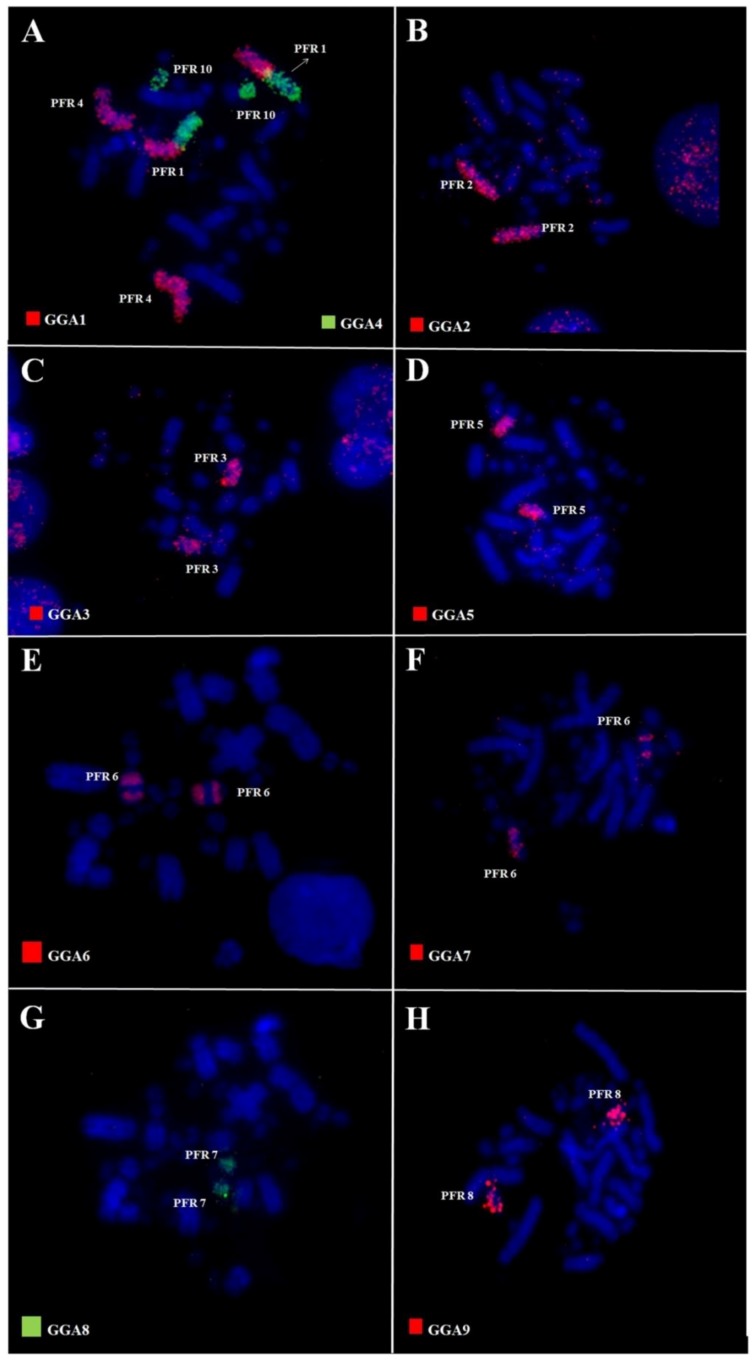
Fluorescent in situ hybridization (FISH) using whole-chromosome probes of *Gallus gallus* 1–9 in *P*. *frontalis*. GGA: *Gallus gallus*; PFR: *Pyrrhura frontalis*.

**Figure 3:**
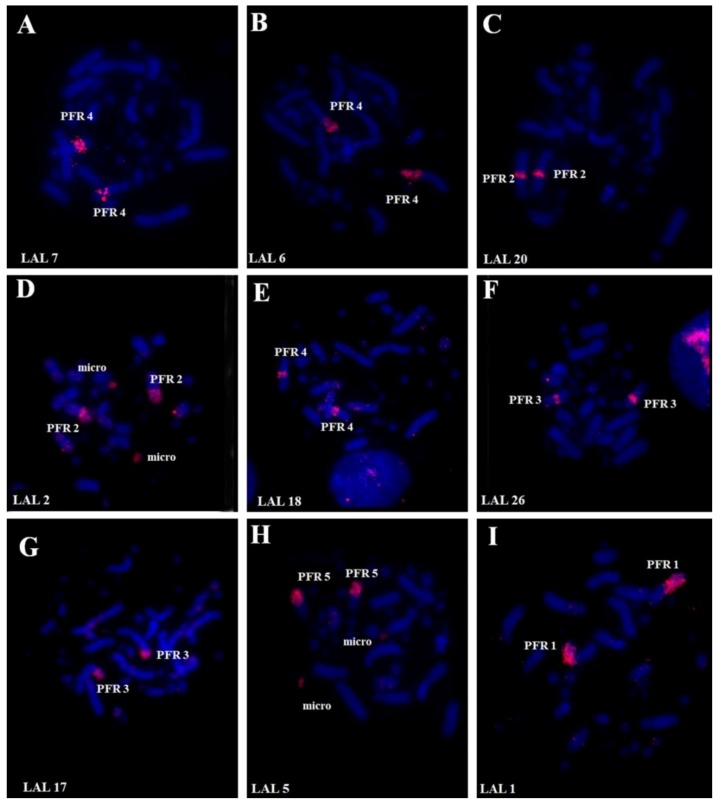
Fluorescent in situ hybridization using whole-chromosome probes of *Leucopternis albicollis* macrochromosomes in metaphases of *P*. *frontalis*. LAL: *Leucopternis albicollis.*

**Figure 4:**
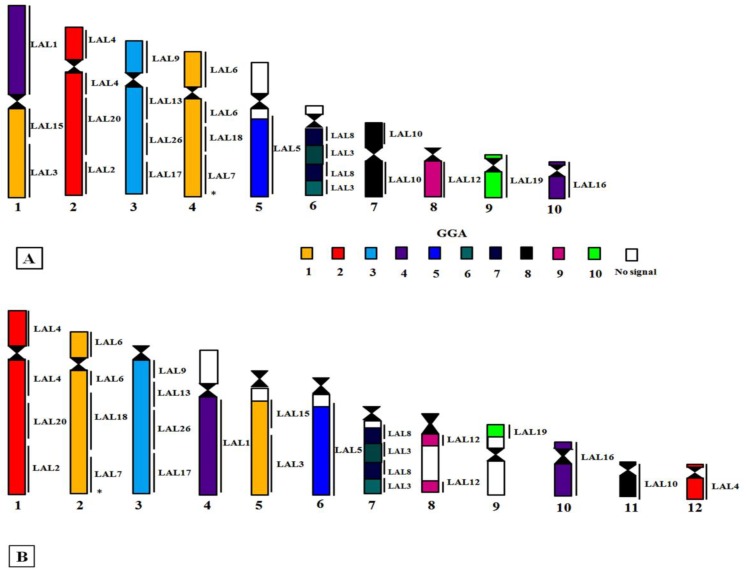
Homology maps with GGA and LAL probes in metaphasic chromosomes of (**A**) *Pyrrhura frontalis* and (**B**) *Amazona aestiva*. Segments not hybridized with LAL probes are indicated with asterisks.

Whole chromosome paints of pairs 1–10 of GGA produced 14 distinct signals in the karyotype of PFR (Figure 2). According to the nomenclature proposed for PAK, we have the following correspondence: PAK1 (GGA1) = PFR1q and PFR4; PAK2 (GGA2) = PFR2; PAK3 (GGA3) = PFR3; PAK4 (GGA4q) = PFR1p; PAK5 (GGA5) = PFR5q; PAK6 (GGA6) = PFR6q; PAK7 (GGA7) = PFR6q; PAK8 (GGA8) = PFR7; PAK9 (GGA9) = PFR8; PAK10 (GGA10) = PFR9, and PAK11 (GGA4p) = PFR10. Hence, we found two fusions in PFR: PFR1 (PAK1q/PAK4) and PFR6 (PAK6/PAK7). In the latter, there was also a paracentric inversion. The use of LAL probes confirmed these results (Figure 3). The resulting homology map of *P*. *frontalis* is shown in Figure 4A.

Furthermore, it was observed that the segment corresponding to GGA1q in both species (PFR4 and AAE2) also shows a gap in the terminal region, suggesting that LAL7 does not cover all this area, and there must be at least one more region of LAL which corresponds to GGA1 not identified by de Oliveira et al. [13], but suggested by Kretschmer et al. [16].

**Figure 5:**
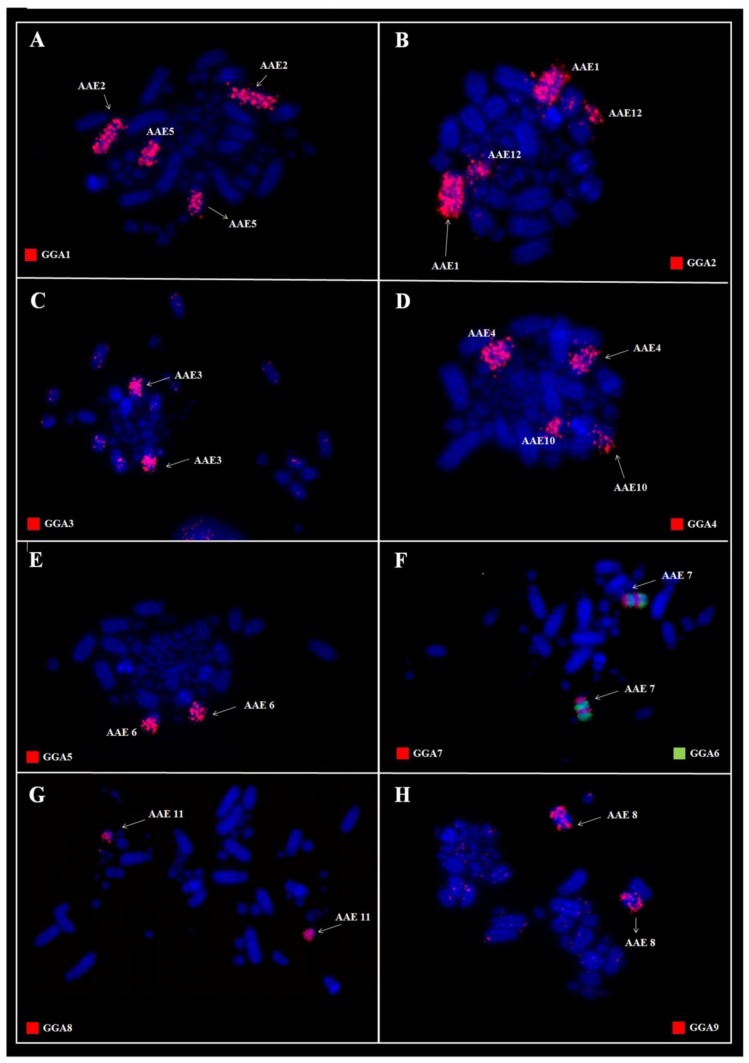
Fluorescent in situ hybridization with whole-chromosome probes of *G. gallus* 1–9 in *A*. *aestiva*. AAE: *Amazona aestiva.*

**Figure 6:**
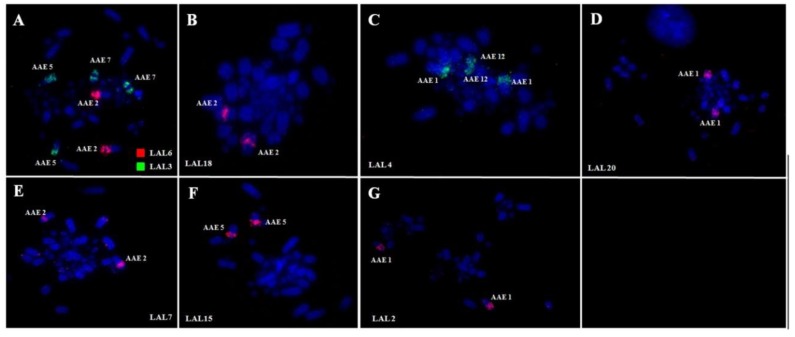
Fluorescent in situ hybridization with whole-chromosome probes of *L. albicollis* in metaphases of *A*. *aestiva*.

In *A*. *aestiva*, we found 16 segments homologous to GGA chromosome paints (Figure 5): PAK1 (GGA1) = AAE2 and AAE5; PAK2 (GGA2) = AAE1 and AAE12; PAK3 (GGA3) = AAE3q; PAK4 (GGA4) = AAE4q; PAK5 (GGA5) = AAE6; PAK6 (GGA6) = AAE7q; PAK7 (GGA7) = AAE7q; PAK8 (GGA8) = AAE11; PAK9 (GGA9) = AAE8; PAK10 (GGA10) = AAE9; PAK11 (GGA4p) = AAE10. In this species, only one fusion was detected: PAK6/PAK7, in AAE7, followed by a paracentric inversion. The experiments using LAL probes confirmed that the breakpoints occurred in syntenic groups according to GGA probes (Figure 6). The homology map of *A*. *aestiva* is shown in Figure 4B.

## 4. Discussion

Cytogenetic studies based on classical cytogenetics have already revealed that parrots, macaws, and related species show considerable chromosomal variability. With the advent of chromosomal painting, this variability has become even more apparent with examples of fusions, fissions, and paracentric inversions.

This was the case in the two species analyzed here, *P*. *frontalis* and *A*. *aestiva*: in comparison with the PAK, our results using GGA and LAL paintings revealed the occurrence of rearrangements involving pairs PAK1, 6, and 7 in both species (Figure 2A,E,F and Figure 5A,F), and PAK2 in *A. aestiva* (Figure 5B and Figure 6C). Additionally, the segment resulting from the fusion of PAK6/PAK7 shows a paracentric inversion in both species. This has also been observed in other species of Neotropical Psittaciformes (*Ara macao*, *Ara chloropterus*, and *Anodorhynchus hyacinthinus*) and in the African grey parrot (*Psittacus erithacus*) [10,11,23].

The presence of the centric fusion of PAK1 in *P*. *frontalis* and *A*. *aestiva* reinforces this rearrangement as a common feature for all the species of Psittaciformes analyzed up to now. Indeed, this centric fission is a homoplasious rearrangement observed in species of different orders of birds, such as Passeriformes, Strigiformes, and Accipitriformes [14,24,25,26]. Additionally, in *P*. *frontalis*, there is also a second rearrangement involving PAK1: the largest element, pair 1 (PFR1), arose by fusion of PAK1p/PAK4 (homologous to GGA1p/GGA4q) (Figure 2A). This rearrangement was observed in *Ara macao*, *Ara chloropterus*, *Anodorhynchus hyacinthinus*, and *Psittacus erithacus* [10,11,23], indicating that it can be considered a cytogenetic signature reinforcing the monophyly of this group. For *Ara macao*, there is also a second fission, and PAK1 has three distinct segments [10].

However, not all the rearrangements are consistent with proposed classifications and biogeography. For instance, a fission of PAK2 (GGA2) is found in *A*. *aestiva* (AAE1 and AAE12). This fission occurred in PAK2p, in the median region of the segment corresponding to LAL4 (Figure 6C). In *A*. *chloropterus* and in *A*. *aestiva*, the same breakpoint was confirmed by the use of LAL probes (a fission occurring in the region corresponding to LAL4). Beyond it, the fission in PAK2 was also observed in *Agapornis roseicollis*, a short-tailed African Psittacidae, however, in this species, the available data include only results obtained with GGA probes, and so it was not possible to determine if the rearrangement happened in the same place.

Other ancestral homologous syntenic blocks that show interesting reorganization in Neotropical Psittaciformes are PAK4 and PAK11 (GGA4q/GGA4p). In most species of birds with data from chromosome painting, these two blocks correspond to two distinct chromosome pairs (although they are fused in *G*. *gallus*) [19,24]. However, in Psittaciformes, although they correspond to two distinct segments, both are found fused to other chromosomes. For instance, species of genera *Ara*, *Anodorhynchus*, *Psittacus*, and *Pyrrhura* show PAK4 (GGA4q) fused to PAK1p (GGA1p) (Table S2). Interestingly, *A*. *aestiva* is the only Neotropical species in which PAK4 (GGA4q) is not fused, as observed in some African and Australian species (Table S2) [10,11,23,27].

Considering biogeographical issues, ancestral syntenic groups PAK6 and PAK7 (GGA6 and GGA7, respectively) are also interesting. These chromosomes are fused in all the Psittaciformes analyzed so far, and in most of them, the newly formed chromosome has undergone a paracentric inversion. This is the situation in Neotropical parrots and macaws, and in the African *Psittacus erithacus* and *A*. *roseicollis* [10,11,23,27]. The fusion PAK6/PAK7 was also reported in Australian Psittaciformes, such as in *Melopsittacus undulatus*, but they do not show any inversion, or the inversion pattern is different to the one described for Neotropical and African species, as in *Agapornis roseicollis* [27] (Table S2). Hence, the fusion PAK6/PAK7 must represent a synapomorphy for Psittaciformes, but the different patterns of inversion and fusions with other segments may have occurred randomly. In addition, the fusion PAK8/PAK9, which was found in all the previously analyzed Psittaciformes, is not found in *P*. *frontalis* and *A*. *aestiva*.

**Figure 7:**
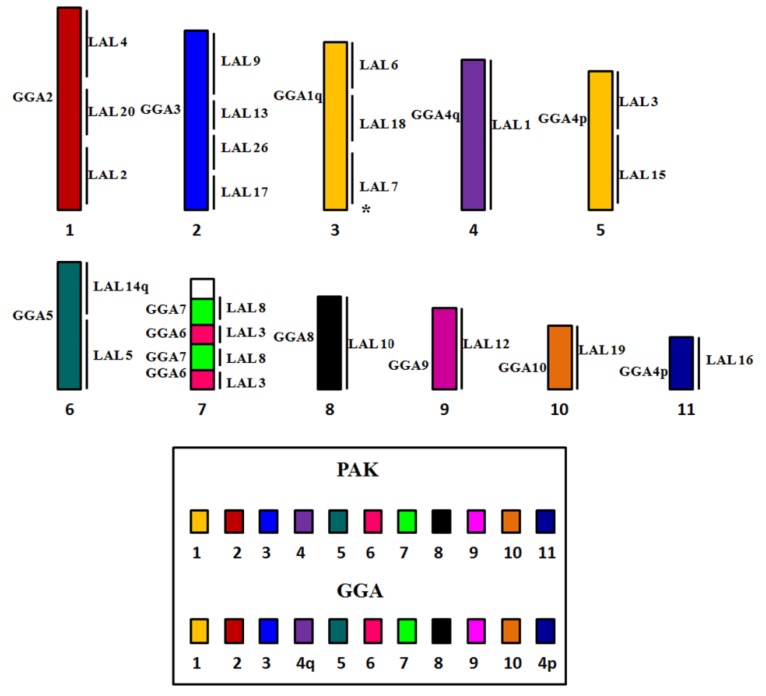
Schematic representation of the PAK of the Tribe Arini, showing fission in PAK 1 and association of PAK6/7, according to FISH results with probes of *L*. *albicollis* and *G*. *gallus* in species of Neotropical Psittacidae. Segments not hybridized with LAL probes are indicated with an asterisk.

Despite the intriguing distribution patterns of these rearrangements in species from different geographical locations, the information generated by chromosome painting allows us to propose a PAK-NP (Figure 7). In our proposal, PAK1 corresponds to two autosomal pairs, due to centric fission, and PAK6/PAK7 are fused and rearranged to form a paracentric inversion. Other macrochromosome pairs correspond to one pair each, as found in the putative avian ancestral karyotype [19,28]. As the karyotypes of Arini species diverged, a fusion of PAK1p/PAK4 appears in the common ancestor of species with a high number of biarmed chromosomes.

### Phylogenetic Analysis

Classification by tail length prior to molecular and cytogenetic studies was used to classify Neotropical Psittacidae into two groups—short- and long-tailed species [29]. This was supported by some minisatellite, behavioral, mtDNA, and karyotypical studies [6,9,30,31]. However, results from more recent studies of mtDNA, involving more genes and a higher number of species, and including genera which were not present in the first analyses, are not in complete agreement with the division of Tribe Arini based on tail length [5].

In a similar way, early cytotaxonomic studies, based on conventional staining, were initially concordant with this division according to tail length. However, as new analyses were performed in a larger number of species, it has become clear that chromosomal morphology sometimes could not support this classification, because there are short-tailed species which show a high number of biarmed macrochromosomes, for example, *Forpus xanthopterygius* (Table S4) [32,33].

The more complete study regarding the phylogenetics of Neotropical Psittacidae had been made by Tavares et al. [5]. According to their studies, this group of birds was divided in three great clades that were described in the introduction of this report, however, our results allowed us to infer, so far, the division only into two groups: long-tailed species (genera *Ara*, *Cyanopsitta*, *Propyrrhura*, *Aratinga*, *Nandayus*, and *Guaruba*), with most macrochromosomes biarmed, and only short-tailed species (genera *Amazona* and *Brotogeris*), with most macrochromosome pairs classified as acro/telocentric [9,22]. However, some species of this tribe have shown karyotypes, such as genus *Pyrrhura* with long tails, transitional between the two formulae mentioned above, with pairs 1 metacentric, pairs 2, 4, and 5 acrocentric, and the remaining macrochromosomes telocentric in *P*. *frontalis* as well as in *P*. *molinae* (PMO), except that in PMO, pair 5 is submetacentric, and pair 2 was classified as metacentric, and pair 1 acrocentric (Table S2) [34]. Hence, although *P*. *frontalis* and *A*. *aestiva* (with the typical karyotype of long- and short-tailed species) showed the same diploid number, 2n = 70, the chromosomal morphology of some pairs differed. Additionally, chromosome painting experiments revealed that their karyotypes are highly divergent, due to the occurrence not only of pericentric inversions, but also of paracentric inversions, fissions, and fusions (Table S4). The analysis of chromosome morphology of species of Arini Tribe suggested that there is probably a group of Psittacidae with intermediate karyotypic characteristics that do not correspond to short- or long-tailed species, which includes genus *Pyrrhura* and *Forpus* (Table S4). However, despite this karyotype transition, and considering the current data, the genus *Pyrrhura* presents chromosomal synapomorphies involving PAK1/4 and PAK8/9, which allow its inclusion in one of the two groups of Neotropical Psittacidae, as discussed below.

**Figure 8:**
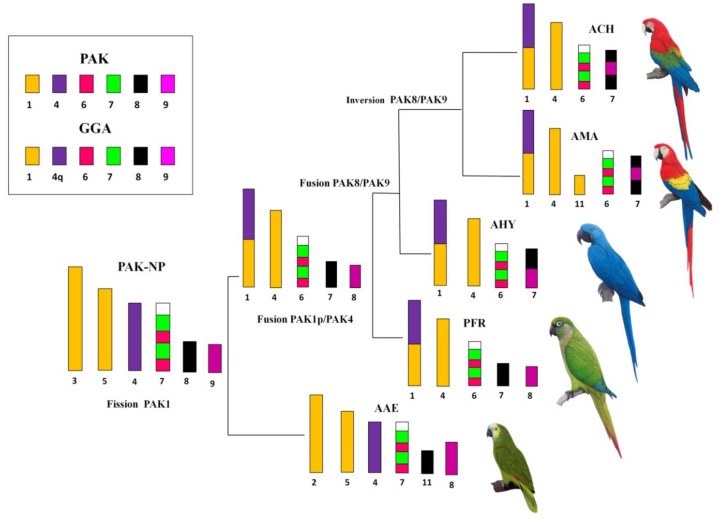
Phylogenetic analysis based on rearrangements involving PAK1, PAK4, PAK6, PAK7, PAK8, and PAK9 in Neotropical Psittacidae, according to results obtained by FISH with probes of *G*. *gallus* and *L*. *albicollis* (legend: PAK, putative ancestral avian karyotype; PAK-NP, putative ancestral karyotype of Neotropical Psittacidae; AMA, *Ara macao*; ACH, *Ara chloropterus*; AHY, *Anodorhynchus hyacinthinus*.

Up at this point, chromosome painting results support the existence of two groups within Arini, based on ancestral chromosomes PAK1p, PAK4, PAK8, and PAK9: one group constituted of species with biarmed chromosomes, in which pair 1 is metacentric, and there are two fusions, PAK1p/PAK4 and PAK8/PAK9, with or without inversions and further fusions. The second group includes species with most macrochromosomes having single arms that show the fission in PAK1, but do not show fusion of PAK8/PAK9, as in genus *Amazona*. Hence, cytotaxomically, this group has a more basal position, corroborating the mtDNA studies [4]. Consequently, species of the first group that share two more synapomorphies, i.e., the PAK1p/PAK4 and PAK8/PAK9 fusions, could be considered more derived. Further rearrangements in PAK8/PAK9 seem to clarify the phylogenetic relationships of some genera, such as *Ara*, *Anodorhynchus*, *Psittacus*, and *Pyrrhura* (Figure 8).

Together with *A*. *aestiva*, *P*. *frontalis* is the only species of Arini which does not have the fusion PAK8/PAK9, which could indicate that both species are more basal than the Neotropical *Ara* and *Anodorhynchus*, and the African *Psittacus*. Additionally, *Anodorhynchus* is more basal than *Ara*, as it has the PAK/8/PAK9 fusion, while *Ara* and *Psittacus* have an additional inversion [11,23]. The similarity between these two genera, despite their divergent geographical distribution, lies in the fact that *Psittacus* is the sister-group of Neotropical Psittacidae. This suggests that African Psittacidae are not a monophyletic group and may have colonized Africa twice—first from Southern Asia, and a second time from South America [35,36].

Nevertheless, although the chromosome data do not allow us to infer about the divergence times among Neotropical Psittacidae, Tavares et al. [5], using molecular data, proposed that the major clades of Neotropical Psittacidae originated about 50 Mya, coinciding with periods of higher sea level when both Antarctica and South America were fragmented with transcontinental seaways and, likely, isolated the ancestors of modern Neotropical parrots in different regions in this continent. However, the diversification of the genus *Ara* occurred around 12 to 13 Mya; in this time, the upheaval of the Andes changed river systems, atmospheric circulation, and rainfall throughout the continent, and altered the geographic distribution of the Neotropical biota to a large extent. The origin of Neotropical parrots—that are today found in open areas, grasslands, and savannah, especially genus *Ara*—coincided with the formation of these habitats in South America. Regarding the same scenario, around 20 Mya, the genus *Anodorhynchus* appeared, while genus *Pyrrhura* appeared around 25 to 23 Mya. Nevertheless, the diversification of the genus *Amazon* occurred around 28 to 24 Mya during the Oligocene, and this “ice house” climate caused a significant drop in sea level during the early Oligocene and caused considerable changes in oceanic and atmospheric circulation.

Although additional studies including other species of Psittacidae are necessary to clarify the phylogeny and biogeography of this group, chromosomal analyses so far suggest that the utilization of the chromosomal synapomorphies, such as associations PAK 1/4, 6/7, and absence of fusion 8/9, could be the best way towards classification of the Neotropical Psittacidae.

## Supplementary Material

The following are available online at http://www.mdpi.com/2073-4425/9/10/491/s1, Table S1: List of specimens collected in the present study, Table S2: Correspondence between syntenic groups of Psittaciformes species analyzed by FISH and the putative ancestral avian karyotype (PAK) and *Gallus gallus* chromosomes (GGA), Table S3: Correspondence between syntenic groups of Psittaciformes species analyzed by FISH with *Gallus gallus* probes, Table S4: Comparison and morphological classification of macrochromosome pairs of 31 species belonging to fourteen genus of Neotropical Psittacidae. A—acrocentric, T—telocentric, SM—submetacentric, MT—metacentric. (−) not described. (*) polymorphic chromosome.
