# Supplementary material for: Chromosome Painting in Neotropical Long- and Short-Tailed Parrots (Aves, Psittaciformes): Phylogeny and Proposal for a Putative Ancestral Karyotype for Tribe Arini
Source: Genes (Basel). 2018 Oct 10;9(10):491. doi: 10.3390/genes9100491 (PMC6210594; doi:10.3390/genes9100491)
Supplement: Supplementary file 1 [file genes-09-00491-s001.zip › genes-348496-supplementary_data_final.docx]

**Table S1.** List of specimens collected in the present study.

| **Species** | **Number** | | **Gender** | **Sample origin** |
| --- | --- | --- | --- | --- |
| *Amazona aestiva* | 2 | 2 ♀ | | Museu Paraense Emílio Goeldi, Belém, Pará |
| *Pyrrhura frontalis* | 2 | 1 ♀ + 1 ♂ | | Parque Zoológico do Rio Grande do Sul, Sapucaia do Sul, Rio Grande do Sul |

**Table S2.** Correspondence between syntenic groups of Psitaciformes species analyzed by FISH and the putative ancestral avian karyotype (PAK) and *Gallus gallus* chromosomes (GGA).

| **Species** | **Chromosomes** | | | | | | | | | | | **2n** | **Distribution** | **References** |
| --- | --- | --- | --- | --- | --- | --- | --- | --- | --- | --- | --- | --- | --- | --- |
| GGA | 1 | 2 | 3 | 4q | 5 | 6 | 7 | 8 | 9 | 4p | 10 | 78 | **-** | [1] |
| PAK | 1 | 2 | 3 | 4 | 5 | 6 | 7 | 8 | 9 | 10 | 11 | 80 | - | [1] |
| AHY | 1q/4 | 2 | 3 | 1p | 5 | 6q | 6q | 7p | 7q | 8q | 9q | 70 | Neotropical | [2] |
| ACH | 1q/4 | 2/11 | 3 | 1p | 5q | 6q | 6q | 7pq | 7q | 8q/10q | 9 | 70 | Neotropical | [2] |
| PFR | 1q/4 | 2 | 3 | 1p | 5q | 6q | 6q | 7 | 8 | 10 | 9 | 70 | Neotropical | [Present work] |
| AMA | 1q/4/9q | 2 | 3 | 1p | 5q | 6q | 6q | 7pq | 7q | 8q | - | 70 | Neotropical | [3] |
| AAE | 2/5q | 1/12 | 3 | 4q | 6 | 7q | 7q | 11 | 8 | 10 | 9p | 70 | Neotropical | [Present work] |
| PER | 1q/4 | 2 | 3 | 4q | 5q | 6q | 6q | 7q | 7q | micro | - | 70 | African | [4] |
| ARO | 3/4q | 2/9q | 1 | 7 | 8q | 6q | 6q | 5q | 5q/9q | 4p | 10 | 48 | African | [5] |
| MUN | 3/6 | 1 | 2 | 7 | 4q | 4p/8p | 4p | 5pq | 5q | 5p | 9q | 62 | Australia | [5] |
| NHO | 3/6 | 1 | 2 | 4 | 7q | 5 | 5 | 4p | 4p/10 | 11 | 9 | 72 | Australia | [5] |

PAK- putative ancestral avian karyotype; AHY- *Anodorhynchus hyacinthinus* / Hyacinth macaw; ACH- *Ara chloropterus* / Red-and-green macaw; PRF- *Pyrrhura frontalis* / maroon-bellied parakeet; AMA- *Ara macao*/ Scarlet macaw; AAE- *Amazona aestiva*/ Turquoise-fronted Parrot; PER- *Psittacus erithacus*/ African grey parrot; ARO- *Agapornis roseicollis*/ Peach-faced lovebird; MUN- *Melopsittacus undulates*/ Budgerigar; MHO- *Nymphicus hollandicus*/ Cockatiel.

**Table S3.** Correspondence between syntenic groups of Psitaciformes species analyzed by FISH with *Gallus gallus* probes.

| **Distribution** | **Species** | **Chromosomes associations** | | | | | | **References** |
| --- | --- | --- | --- | --- | --- | --- | --- | --- |
|  |  | 1q/4 | 6/7 | 8/9 | 4/8/9 | 5/6/7 | 2/9 |  |
| Neotropical Psittacidae | *Anodorhynchus hyacinthinus* | * | * | * |  |  |  | [2] |
|  | *Ara chloropterus* | * | * | * |  |  |  | [2] |
|  | *Ara macao* | * | * | * |  |  |  | [3] |
|  | *Pyrrhura frontalis* | * | * |  |  |  |  | [Present study] |
|  | *Amazona aestiva* |  | * |  |  |  |  | [Present study] |
| Australian Psittacidae | *Melopsittacus undulatus* |  | * |  | * |  |  | [5] |
|  | *Nymphicus hollandicus* |  |  |  | * | * |  | [5] |
| African Psittacidae | *Agapornis roseicollis* |  | * | * |  |  | * | [5] |
|  | *Psittacus erithacus* | * | * | * |  |  |  | [4] |

**Table S4.** Comparison and morphological classification of macrochromosome pairs of 31 species belonging to fourteen genus of Neotropical Psittacidae. A-acrocentric, T- telocentric, SM-submetacentric, MT-metacentric. (-) not described. (*) polymorphic chromosome.

| **Genus** | **Species** | **1** | **2** | **3** | **4** | **5** | **6** | **7** | **8** | **9** | **10** | **References** | **Tail size** |
| --- | --- | --- | --- | --- | --- | --- | --- | --- | --- | --- | --- | --- | --- |
| *Anodorhynchus* | *Anodorhynchus leari* | MT | SM | SM | SM | SM | SM | MT | M | SM | MT | [6] | Long tail |
| *Anodorhynchus* | *Anodorhynchus hyacinthinus* | MT | A | A | A | SM | SM | MT | SM | SM | MT | [7] | Long tail |
| *Ara* | *Ara chloroptera* | MT | A | SM | A | SM | A | MT | MT | T | MT | [8] | Long tail |
| *Ara* | *Ara ararauna* | MT | A | SM | A | SM | A | MT | MT | T | MT | [8] | Long tail |
| *Ara* | *Ara macao* | MT | A | SM | A | SM | A | MT | MT | T | MT | [8] | Long tail |
| *Propyrrhura* | *Propyrrhura maracana* | MT | A | A | A | SM | A | MT | MT | T | MT | [8] | Long tail |
| *Nandayus* | *Nandayus nenday* | MT | A | SM | A | SM | A | MT | MT | T | MT | [8] | Long tail |
| *Aratinga* | *Aratinga auricapilla* | MT | A | SM | A | SM | A | MT | MT | T | MT | [8] | Long tail |
| *Aratinga* | *Aratinga guarouba* | MT | A | SM | A | A | A | SM | MT | MT | - | [9] | Long tail |
| *Aratinga* | *Aratinga acuticaudata* | MT | A | SM | A | A | A | SM | MT | MT | - | [9] | Long tail |
| *Aratinga* | *Aratinga finschi* | MT | A | A | A | A | A | A | MT | MT | T | [10] | Long tail |
| *Deroptyus* | *Deroptyus accipitrinus* | MT | A | A | A | A | SM | MT | MT | T | T | [7] | Long tail |
| *Myiopsitta* | *Myiopsitta monachus* | MT | SM | MT | MT | MT | SM | MT | MT | MT | MT | [11] | Long tail |
| *Pyrrhura* | *Pyrrhura frontalis* | MT | A | * | A | A | T | T | T | T | T | [In this study] | Long tail |
| *Pyrrhura* | *Pyrrhura molinae* | A | MT | A | A | SM | T | T | T | T | T | [8] | Long tail |
| *Triclaria* | *Triclaria malachitacea* | MT | A | A | A | A | A | MT | MT | MT | MT | [10] | Long tail |
| *Forpus* | *Forpus xanthopterygius* | MT | MT | T | A | MT | T | T | T | - | - | [12] | Short tail |
| *Pionus* | *Pionus maximiliani* | A | A | A | A | A | T | T | - | - | - | [13] | Short tail |
| *Pionus* | *Pionus mestruus* | A | A | A | A | A | T | T | - | - | - | [8] | Short tail |
| *Pionus* | *Pionus* seniloides | A | A | A | A | T | A | T | - | - | - | [8] | Short tail |
| *Graydidascalus* | *Graydidascalus brachyurus* | A | A | A | A | A | A | MT | MT | - | - | [13] | Short tail |
| *Salvatoria* | *Salvatoria xanthops* | A | SM | SM | SM | SM | T | MT | MT | - | - | [14] | Short tail |
| *Amazona* | *Amazona aestiva* | T | SM | A | SM | T | T | T | T | MT | A | [11] | Short tail |
| *Amazona* | *Amazona amazonica* | T | A | A | SM | T | T | T | MT | - | - | [14] | Short tail |
| *Amazona* | *Amazona brasiliensis* | T | SM | A | SM | T | T | T | MT | - | - | [14] | Short tail |
| *Amazona* | *Amazona festiva* | T | A | SM | SM | T | T | T | MT | - | - | [14] | Short tail |
| *Amazona* | *Amazona kawalli* | T | SM | A | SM | T | T | T | MT | - | - | [14] | Short tail |
| *Amazona* | *Amazona ochrocephala* | T | A | SM | SM | T | T | T | MT | - | - | [14] | Short tail |
| *Amazona* | Amazona rhodocorytha | T | SM | A | SM | T | T | T | MT | - | - | [14] | Short tail |
| *Amazona* | *Amazona farinosa* | T | SM | SM | SM | T | T | T | MT | - | - | [14] | Short tail |
| *Amazona* | Amazona vinacea | T | SM | SM | SM | T | T | T | MT | - | - | [14] | Short tail |

References

1. Griffin, D.K.; Robertson, L.B.; Tempest, H.G.; Skinner, B.M. The evolution of the avian genome as revealed by comparative molecular cytogenetics. *Cytogenet. Genome Res.* **2007**, *117*, 64–77.
2. Furo, I.O.; Kretschmer, R.; O’Brien, P.C.M.; Ferguson-Smith, M.A.; de Oliveira, E.H.C. Chromosomal diversity and karyotype evolution in South American macaws (Psittaciformes, Psittacidae). *PLoS ONE* **2015**, *10*, e0130157.
3. Seabury, C.M.; Dowd, S.E.; Seabury, P.M.; Raudsepp, T.; Brightsmith, D.J.; Liboriussen, P.; Halley, Y.; Fisher, C.A.; Owens, E.; Viswanathan, G.; et al. A multiplatform draft de novo genome assembly and comparative analysis for the scarlet macaw (*Ara macao*). *PLoS ONE* **2013**, *8*, e62415.
4. Seibold-Torres, C.; Owens, E.; Chowdhary, R.; Ferguson-Smith, M.A.; Tizard, I.; Raudsepp, T. Comparative cytogenetics of the Congo African grey parrot (*Psittacus erithacus*). *Cytogenet. Genome Res.* **2016**, *147*, 144–153.
5. Nanda, I.; Karl, E.; Griffin, D.K.; Schartl, M.; Schmid, M. Chromosome repatterning in three representative parrots (Psittaciformes) inferred from comparative chromosome painting. *Cytogenet. Genome Res.* **2007**, *117*, 43–53.
6. Nogueira, D.M.; Souza, L.M.; Goldschmidt, B.; Silva, C.P.; Monsores, D.W. The karyotype of the critically endangered Lear’s macaw, *Anodorhynchus leari* Bonaparte 1856 (Aves,Psittaciformes). *Genet. Mol. Biol.* **2006**, *29*, 656–658.
7. Lunardi, V.O.; Francisco, M.R.; Rocha, G.T.; Goldschmidt, B.; Galetti, J.P.M. Karyotype description of two Neotropical Psittacidae species: The endangered Hyacinth macaw, *Anodorhynchus hyacinthinus*, and the Hawk-headed Parrot, *Deroptyus accipitrinus* (Psittaciformes: Aves), and its significance for conservation plans. *Genet. Mol. Biol.* **2003**, *26*, 283–287.
8. Francisco, M.R.; Galetti, J.P.M. Cytotaxonomic considerations on Neotropical Psittacidae birds and description of three new Karyotypes. *Hereditas* **2001**, *134*, 225–228.
9. Goldschmidt, B.; Nogueira, D.M.; Mansores, D.W.; Souza, L.M. Chromosome study in two Aratinga species (*Aratinga guarouba* and *Aratinga acuticauda*) (Psittaciformes). *Braz. J. Genet.* **1997**, *20*, 659–662.
10. Rus, A.V.; Cigudosa, J.C.; Juan, C.; Gómez, A.O.; Almeida, T.A.; Joshua, S.; Miranda, J.L.G. Chromosomal Evolution in Psittaciformes. *Int. J. Biol.* **2016**, *8*, 34–65.
11. Furo, I.O.; Kretschmer, R.; dos Santos, M.S.; Carvalho, C.A.L.; Gunski, R.J.; O’Brien, P.C.M.; Ferguson-Smith, M.A.; Cioffi, M.B.; de Oliveira, E.H.C. Chromosomal mapping of repetitive DNAs in *Myiopsitta monachus* and *Amazona aestiva* (Psittaciformes, Psittacidae: Psittaciformes), with emphasis on the sex chromosomes. *Cytogenet. Genome Res.* **2017**, *151*, 151–160.
12. de Lucca, EJ.; de Marco, D.A. Chromosomal Polymorphism in *Forpus Xanthopterygius* (Psittaciformes: Aves). *Caryologia* **1983**, *36*, 355–361.
13. Caparroz, R.; Duarte, J.M.B. Chromosomal similarity between the Scaly-headed parrot (*Pionus maximiliani*), the Short-tailed parrot (*Graydidascalus brachyurus*) and the Yellow-faced parrot (*Salvatoria xanthops*) (Psittaciformes: Aves): A cytotaxonomic analysis. *Genet. Mol. Biol.* **2004**, *27*, 522–528.
14. Duarte, J.M.B.; Caparroz, R. Cytotaxonomic analysis of Brazilian species of the genus *Amazona* (Psittacidae, Aves) and confirmation of the genus *Salvatoria* (Ribeiro,1920). *Braz. J. Genet.* **1995**, *18*, 623–628.

© 2018 by the authors. Submitted for possible open access publication under the terms and conditions of the Creative Commons Attribution (CC BY) license (http://creativecommons.org/licenses/by/4.0/).
